# Creation of Superhydrophobic and Superhydrophilic Surfaces on ABS Employing a Nanosecond Laser

**DOI:** 10.3390/ma11122547

**Published:** 2018-12-14

**Authors:** Cristian Lavieja, Luis Oriol, José-Ignacio Peña

**Affiliations:** 1 Instituto de Ciencia de Materiales de Aragón, Universidad de Zaragoza-CSIC Dpto. Ciencia y Tecnología de Materiales y Fluidos Maria de Luna 3, 50018 Zaragoza, Spain; clavieja@unizar.es; 2 Instituto de Ciencia de Materiales de Aragón, Universidad de Zaragoza-CSIC Dpto. Química Orgánica-Facultad de Ciencias Pedro Cerbuna 12, 50009 Zaragoza, Spain; loriol@unizar.es

**Keywords:** nanosecond laser surface modification, ABS (Acrylonitrile-Butadiene-Styrene), surface wettability, superhydrophobic, superhydrophilic

## Abstract

A nanosecond green laser was employed to obtain both superhydrophobic and superhydrophilic surfaces on a white commercial acrylonitrile-butadiene-styrene copolymer (ABS). These wetting behaviors were directly related to a laser-induced superficial modification. A predefined pattern was not produced by the laser, rather, the entire surface was covered with laser pulses at 1200 DPI by placing the sample at different positions along the focal axis. The changes were related to the laser fluence used in each case. The highest fluence, on the focal position, induced a drastic heating of the material surface, and this enabled the melted material to flow, thus leading to an almost flat superhydrophilic surface. By contrast, the use of a lower fluence by placing the sample 0.8 µm out of the focal position led to a poor material flow and a fast cooling that froze in a rugged superhydrophobic surface. Contact angles higher than 150° and roll angles of less than 10° were obtained. These wetting behaviors were stable over time.

## 1. Introduction

The wettability of solid surfaces is of particular interest because of the wide range of applications derived from the control of this property, which is directly related to the surface structure or roughness, and the chemical interactions with a liquid. Nature has provided some examples of structures that affect the wettability properties, such as self-cleaning superhydrophobic surfaces on the Lotus leaf [1], hierarchical structures on plants [2], butterflies that exhibit directional wettability properties [3], or the adhesion properties of the Gecko toe [4]. The surface interaction of a water drop deposited on a surface is characterised by a contact angle (CA). This angle, θ is measured between the solid/liquid and liquid/air interfaces. A surface is considered to be hydrophilic if its static CA is below 90°, and superhydrophilic if this CA is lower than 10°. By contrast, surfaces with a static CA higher than 90° are hydrophobic, and if the CA is higher than 150°, the surface can be considered as superhydrophobic [5]. Additionally, a non-equilibrium situation is measured by defining the advancing and receding angle of a drop sliding on a vertical surface. The contact angle hysteresis (CAH) measures the difference between the two angles. A low CAH is exhibited by a surface on which the drop descends easily. In contrast, a surface with a relatively high CAH has a strong interaction with the drop (i.e., the drop tends to adhere to the surface) [6,7]. The roughness of the surface influences the wettability according to either the Wenzel model [8], where the liquid fills all of the space, or the Cassie-Baxter model, which considers that some air bubbles are trapped between the drop and the surface [9].

Control of the wettability properties of materials is widely studied and applied in numerous fields [10]. For example, there is special interest in surfaces that have self-cleaning properties [11], anti-frosting and ice-resistance behavior [12,13], anti-fouling [14,15], anti-corrosion [16,17], or transparent and anti-reflective properties [18,19]. Different chemical coatings or physical patterning processes have been used to mimic surfaces with these properties, especially on polymeric materials. The proposed methods usually work properly at a laboratory scale, but are challenging when implemented for mass production. However, in the last two decades, an increasing number of techniques have demonstrated the potential of scale-up material processing for the control of surface properties [20]. One interesting industrial strategy to change the surface topography is the use of a laser beam as a micromachining tool [21].

Lasers have been widely used to tune the wettability properties of materials like metal [22,23,24,25,26,27], silicon [28,29], or different polymeric materials [30,31,32]. Various approaches have been employed to change polymer wetting behavior using lasers, in particular, the chemical activation of the surface [33], controlled isomerisation of photoresponsive moieties [34], or nano- and micro-patterning [35,36,37,38]. Different strategies can be studied to modify the material surface in a controlled way using a patterning approach [39]. For example, ultra-short lasers are used to produce very precise ablation, thus reducing the thermal impact and enabling highly defined patterns [40,41] or the creation of ripples [42,43]. However, the use of these lasers does not extend to the manufacturing industry, because of their high cost. Other proposed techniques involve processes like beam shaping control [44] or micro-machining of the injection mold [45,46]. The use of nanosecond pulse-width lasers to modify the wettability of polymers, particularly ABS, has not been widely reported. These lasers have the advantage of a low cost compared with the ultra-short lasers. In the work described here, a nanosecond green laser was used to modify the roughness of a commercial ABS—and thus its wetting properties—in order to obtain both superhydrophobic and superhydrophilic behaviour.

## 2. Materials and Methods

The laser used was a Nd:YVO_4_ nanosecond laser (Trumark 6230 from Trumpf, Ditzingen, Germany). The laser generates a gaussing beam with no defined polarization at a wavelength of 532 nm. A 100 mm lens was used to provide a beam spot size with a focus of 30 µm. The pulse width was 8 ns, operating at a pulse frequency of 15 kHz. The power of the laser was 3.5 W, providing a fluence in focus of 34.4 J/cm^2^, that is, far above the ablation threshold of the ABS, which is 0.3 J/cm^2^ [47]. The laser source was attached to a motorised arm that was able to move the system in the vertical axis with micro-scale precision. Furthermore, the system had a beam scanner controlled by CAD software (TruTops Mark 2.6).

The material selected was a white commercial ABS employed in the automotive and home appliance industries (Elix P2H-AT, ELIX Polymers, La Pineda, Spain); in particular, in this last industrial sector, the control of the wettability properties can be of interest for different applications. The ABS samples were cleaned with isopropanol prior to irradiation. The material surface was analyzed using a confocal microscope (Sensofar Plu 2300, Barcelona, Spain), which allows for 3D images to be obtained as well as for the measurement of the physical dimensions of the superficial structures. Environmental scanning electron microscopes (ESEM, QUANTA FEG 250, Thermo Fisher Scientific, Waltham, MA, USA) was also used to provide information about the topography. Information of the chemical modification after laser treatment was obtained by employing X-ray photoelectron spectroscopy (XPS). The equipment was a Kratos AXIS Ultra DLD (Manchester, UK, Mono Al Kα, Power: 120 W (10 mA, 15 kV)). The binding energies were calibrated using the C 1s signal (284.6 eV) of adventitious carbon. The wettability of the material and the stability of the wetting properties were measured using a contact angle goniometer (Theta Lite Optical Tensiometer from Biolin Scientific, Gothenburg, Sweden). The samples were stored under controlled environmental conditions for at least six months (temperature ~22 °C and relative humidity ~40%, UV light (below ~400 nm) was avoided, and special protection against dust was not taken).

## 3. Results and Discussion

The topography and wetting properties of the material can be tuned by irradiation and by controlling the laser parameters such as the overlap between the pulses, the number of spots per area (DPI), and/or the laser fluence over the material [38,48]. The study described here was carried out at a constant value of 1200 DPI (consequently, the total energy deposited on the surface was constant), and the fluence of each laser pulse was modified by placing the sample in different positions along the focal axis. Specifically, a distance of 2 mm up and down out of the focal plane in successive steps of 0.1 mm was employed. Thus, the fluence ranged from 5 to 35 J/cm^2^. As a consequence of this approach, the overlap of the laser pulses changed depending on the sample position.

### 3.1. Topographical Characterization

**Figure 1:**
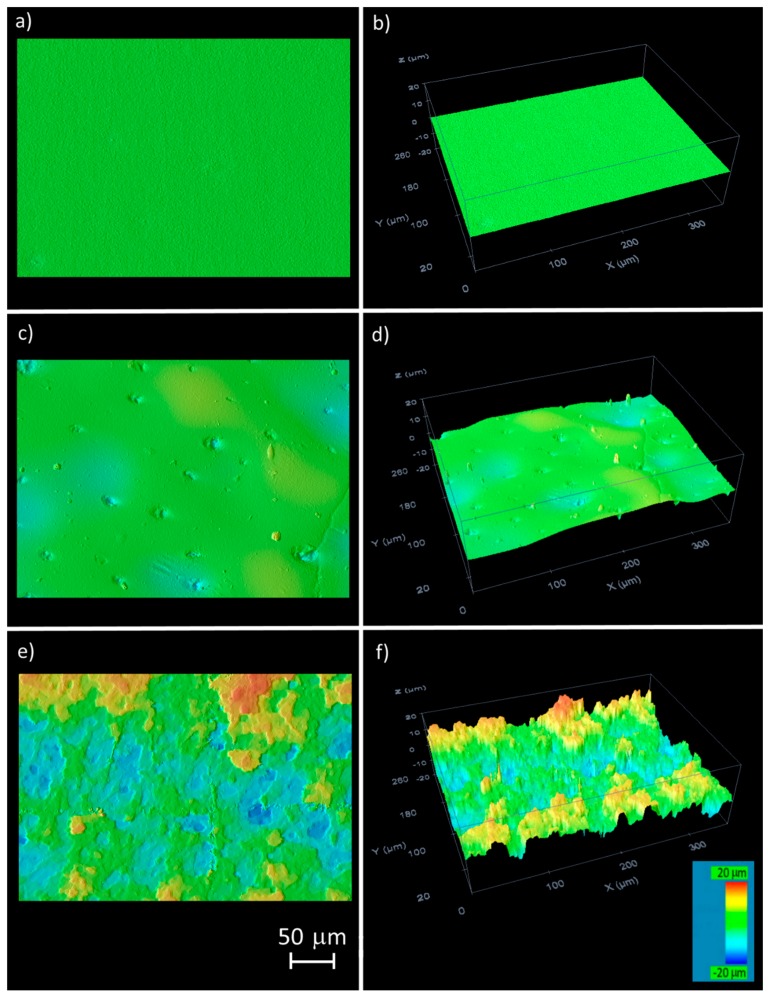
Topographical images of the ABS surface roughness observed by confocal microscopy: (**a**,**b**) before laser irradiation; (**c**,**d**) after green laser irradiation in focus; (**e**,**f**) after green laser irradiation 0.8 mm out of focus.

The material surface was studied by confocal microscopy before and after the laser treatment. Two types of topography were created, depending on the deposited fluence. Although a clear transition was not detected, a change was observed when the samples were irradiated at around 0.6 mm out of focus. The two general types of generated surfaces corresponding to the irradiation in focus and ±0.8 mm out of the focal position (the results were similar in both directions) are shown in Figure 1, together with the unmarked material. The surface prior to marking was flat (Figure 1a,b). After green laser treatment in focus (Figure 1c,d), the topography that was detected was characterised as being almost flat, although small ripples could be observed (changes from green to pale blue in Figure 1c,d). The presence of some small holes was also observed. Thus, the total surface area had increased slightly in comparison to the unmarked material. By contrast, the ABS treated ±0.8 mm out of the focal position (Figure 1e,f) had a rugged topography characterized by the presence of peaks and valleys in the irradiated area. In this case, the increase in the surface area was significantly higher than in the previous case. 

**Figure 2:**
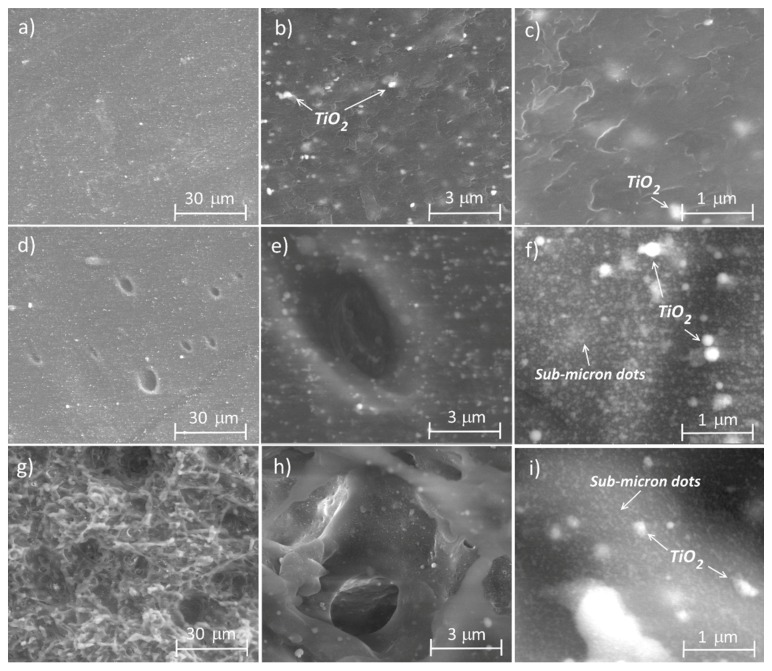
ESEM images of the ABS surface roughness: (**a**–**c**) before laser irradiation; (**d**–**f**) after green laser irradiation in focus; (**g**–**i**) after green laser irradiation 0.8 mm out of focus. Magnification: (**a**) 2800× scale bar 30 µm; (**b**) 24,000×, scale bar 3 µm; (**c**) 80,000×, scale bar 1 µm.

A more detailed image was obtained using ESEM. The images taken by this technique on the unmarked material surface and of the materials marked in focus and 0.8 mm out of focus are presented in Figure 2 (at three different magnifications). Once again, clear differences between the analyzed surfaces were observed. The unmarked material (Figure 2a–c) exhibited a smooth surface. White dots were visible in the image taken at 24,000 × magnification, and these mainly correspond to the TiO_2_ particles (used as a whitening additive), according to the EDX (Energy Dispersive X-ray spectroscopy) analysis (the intensity of the Ti signal was around three times higher than in the rest of the material). When the surface was observed at the highest magnification, a wavy area was evident, probably due to the injection process. The ABS marked in focus (Figure 2d–f) also had a smooth surface. However, as observed by confocal microscopy, this surface had some holes distributed throughout the area. The presence of white dots corresponding to the TiO_2_ particles was also observed. In addition, the appearance of a sub-micron dotted structure was detected using the highest magnification, probably associated with the laser treatment. The diameter of these points was less than 100 nm. Finally, the surface of the material marked 0.8 mm out of focus (Figure 2g–i) had a rugged topography. The surface was characterised by the presence of peaks in all directions. A slight tilting of the samples clearly showed that the surface was chaotic and that holes and channels had formed in some of the areas. These areas may be associated with the material filaments that were melted during the laser treatment, and then rapidly solidified. The sub-micron structure of the points was also visible in this sample. Finally, the presence of white points associated with the TiO_2_ particles was also detected, although these were difficult to observe because of the high roughness of the surface.

These changes can be explained by the fluence differences on the surface. The highest fluence used (i.e., in focus) causes the heating of the material, which leads to a quasi-pure ablation effect together with the formation of some melted surface. The material was heated up very rapidly and it then began to flow before cooling down, which led to bubbles being trapped within the material, giving the appearance of small holes. By contrast, on positioning the sample out of focus, the temperature reached at the surface was lower and the temperature of the material was not sufficient to achieve flow before it dropped again. Furthermore, the laser generated additional pressure and this led to bubble formation and the ejection of the material. The cooling of the surface caused the freezing of this rugged structure.

**Figure 3:**
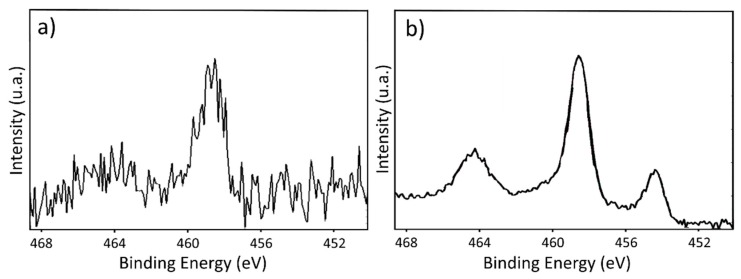
XPS high-resolution spectrum of the Ti region of the ABS: (**a**) before laser irradiation; (**b**) after green laser irradiation.

In addition to the topographical properties, an XPS analysis was carried out to study the chemical changes on the ABS surface after the laser irradiation. No significant modification on the total percentages of the total atomic percentages was detected after the marking process. However, the signal intensity corresponding to the Ti peak, detected at 455 eV, was higher (around five times) compared with the other peaks after the laser treatment. High-resolution experiments of the region of the binding energies correspond to Ti 2p (Figure 3) showed changes after the laser treatment, while negligible differences were observed between the treated surfaces on and out of focus. Two main peaks were detected, at 459 eV and at 464 eV (although this one is barely visible in the unmarked sample) corresponding to Ti^4+^ 2p^3/2^. After the laser irradiation (Figure 3b), another peak at lower binding energies was detected, around 454 eV. According to the literature, this peak can be ascribed to TiC [49,50,51]. No significant evidence of other peaks after the laser treatment were detected on the analysed samples.

### 3.2. Wetting Properties

The wetting properties of the irradiated materials were studied by employing a contact angle goniometer. The contact angles cannot be simulated because of the chaotic topography of the structures created. As a result of the high CA obtained in some cases, the CAH was also measured. For this purpose, the plane containing the droplet was tilted, and the advancing and receding contact angles (θ_A_ and θ_R_) were measured when the droplet started to slide down [7]. The CA of the unmarked material was 62.8 ± 1.7°.

The most significant results were obtained after the surface had been processed with the laser in terms of the position along the focal axis, and the corresponding fluence is shown in Table 1. Although only two differentiated structures were observed using confocal microscopy, three different wetting behaviors were detected. The samples marked close to the focus (less than ±0.5 mm from the focal position) exhibited superhydrophilic behaviour [52]. The water drop wetted the entire surface, and, consequently, the CA was considered to be lower than 10°. By contrast, when the material was treated slightly further away from the focus (±0.8 mm or ±0.9 mm), dramatic changes in the wetting properties of the surface were observed. The CA increased to values higher than 150°, and the surface was considered to show superhydrophobic behavior [52]. Furthermore, the measured CAH was less than 10°; the drop did not adhere to the surface and tended to slide when the plane was slightly tilted. Finally, the samples marked out of the focal position by more than ±1.2 mm also exhibited a high CA value (i.e., close to superhydrophobic behaviour), although in this case, the drop was pinned to the surface and did not slide down upon tilting, even to the vertical position. The temporal evolution of the CA was studied for a period of six months, but significant changes were not detected in the samples.

**Figure 4:**
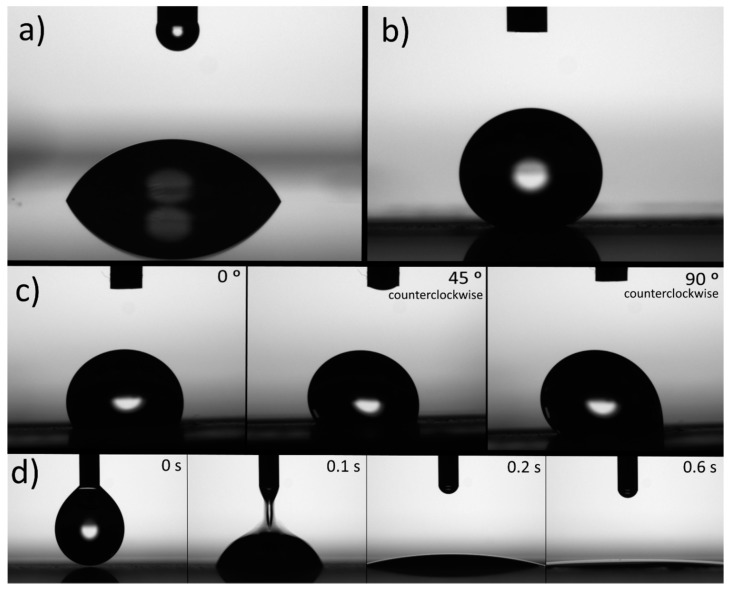
Images of a water drop deposited on ABS: (**a**) untreated surface; (**b**) surface irradiated by laser 0.9 mm out of focus; (**c**) surface irradiated by laser 1.3 mm out of focus and taken at different tilt angles (the camera rotated simultaneously with the surface); (**d**) time-lapse images of a drop on the surface treated by the laser in focus.

The images of the water drops on the ABS surfaces irradiated with the green laser and observed by the contact angle goniometer camera are shown in Figure 4. The behavior of a water drop deposited on the untreated material is shown in Figure 4a as a reference. Figure 4b corresponds to a surface treated with the green laser 0.9 mm out of the focal position, and this image represents superhydrophobic behavior. The behavior of a water drop on the surface marked 1.3 mm out of focus is shown in Figure 4c, and the images were taken at different tilt angles (0°, 45°, and 90°). The water drop exhibited a high adhesion and was pinned to the surface, as can be seen for a tilt angle of 90°. Finally, time-lapse images of a drop on the surface marked by the laser in focus are shown in Figure 4d. The water was dispersed on the surface within one second, thus revealing superhydrophilic behavior. A slow-motion video sequence of water drops falling on the superhydrophilic and on the superhydrophobic surfaces is provided in the Supplementary Materials.

**Figure 5:**
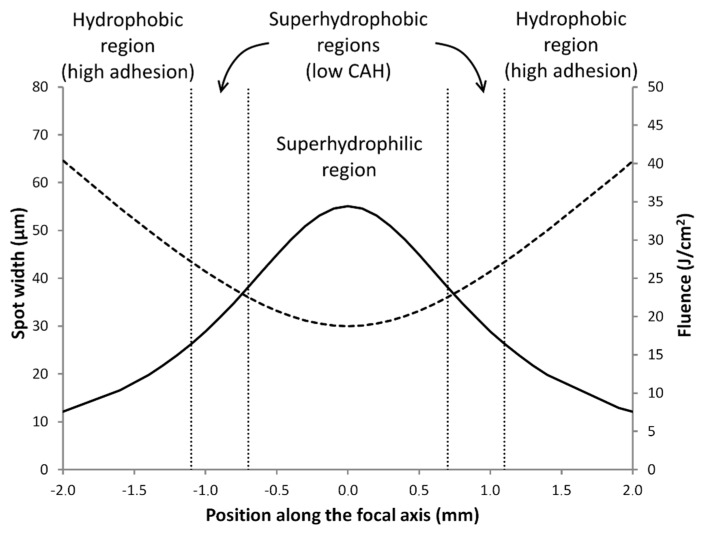
Different wetting behaviors in terms of the sample position along the focal axis compared with the spot width (dashed line, units on the left axis) and fluence (continuous line, units on the right axis) of the green laser.

It is clear that the wettability of the ABS can be changed by irradiation with a green laser, and three different wetting behaviors can be obtained depending on the irradiation conditions, as follows: a region of superhydrophilic behavior and two regions characterised by high CAs, one associated with low CAH and the other one exhibiting a high adhesion to the surface. These different behaviors (Figure 5) can be achieved depending on the sample position along the focal axis and the corresponding spot width and fluence of the green laser. The superhydrophilic region was associated with the focal position. The topography observed (Figure 1c,d) was characterised as being almost flat. This region is characterised by the highest fluence values (i.e., above 25 J/cm^2^). The surfaces treated in the transition zone to the adjacent region showed unstable behaviour; initially, the surface seemed to be hydrophobic, but under a small perturbation (or after several seconds), the drop began to wet the surface in a manner consistent with superhydrophilic behavior, probably because of the inhomogeneous topography observed. The adjacent region showed a high CA together with a low CAH. Also, the fluence associated with this region was less than in the case of the superhydrophilic region, and the laser fluences in the approximate range between 15 and 25 J/cm^2^ seemed to produce this behavior. The transition samples to the next region displayed heterogeneous behavior, in which adhered and non-adhered drops were observed on the same marked surface. Finally, the last region showed hydrophobic behavior and a high CAH. The lowest fluence studied gave rise to this behavior, namely values below 15 J/cm^2^.

It is possible to correlate the topography of the surfaces analysed with their wetting properties. The superhydrophobic surface (Figure 2g) had a rugged topography with numerous peaks and holes, which caused a reduction in the top material surface area. Thus, following the Cassie-Baxter approach, this reduction leads to an increase in the CA, and superhydrophobic behaviour is observed. Furthermore, the water did not adhere to the material and it slid easily upon the surface. By contrast, the material that presented a high CA but with a high hysteresis behaviour also had a rough surface, but with a lower peak density. In this case, the water was able to penetrate slightly into the topography, thus leading to a high adhesion to the surface. Finally, the superhydrophilic surface (Figure 2d) had a structure characterised by smooth hills, which increased the ratio of the surface area when compared to the unmarked material. According to the Wenzel approach, the CA, in this case, should be lower than that for the untreated surface.

## 4. Conclusions

The use of a green laser in the range of nanosecond pulses provided an effective method to control the final wettability of ABS, with both superhydrophilic and superhydrophobic behaviors being observed. These wetting properties were reliant on the surface topography created, and this was directly related to the laser parameters such as the fluence. Three types of topographies were created—one smooth surface that exhibited superhydrophilic behavior and two surfaces with a rugged topography that showed superhydrophobic behavior, one with a low CAH and the other with a high adhesion effect. No significant chemical differences were detected between the marked samples that could affect to the wetting properties. All of the structures were stable over time.

## Figures and Tables

**Table 1:** The CA and CAH measured on some of the structures created with the green laser.

Position Respect to Focus (mm)	Fluence (J/cm^2^)	Overlap between Pulses	Topography Observed	CA	CAH
0	34.4	29.4	smooth	<10°	-
±0.4	30.1	34.1	smooth	<10°	-
±0.6	26.0	38.7	slightly rugged	unstable	-
±0.8	21.8	43.9	rugged	168 ± 3°	<10°
±0.9	19.9	46.4	rugged	170 ± 3°	<10°
±1.2	14.9	53.5	rugged	153 ± 5°	pinned
±1.3	13.6	55.7	rugged	143 ± 5°	pinned

## Supplementary Materials

The following are available online at http://www.mdpi.com/1996-1944/11/12/2547/s1, Video: A slow-motion video sequence of water drops falling on the superhydrophilic and on the superhydrophobic surfaces.
